# Inhibition of Spleen Tyrosine Kinase Restores Glucocorticoid Sensitivity to Improve Steroid-Resistant Asthma

**DOI:** 10.3389/fphar.2022.885053

**Published:** 2022-05-05

**Authors:** Qian Liu, Lijuan Hua, Chen Bao, Luxia Kong, Jiannan Hu, Chao Liu, Ziling Li, Shuyun Xu, Xiansheng Liu

**Affiliations:** ^1^ Department of Respiratory and Critical Care Medicine, NHC Key Laboratory of Respiratory Diseases, Key Site of National Clinical Research Center for Respiratory Disease, Wuhan Clinical Medical Research Center for Chronic Airway Diseases, Tongji Hospital, Tongji Medical College, Huazhong University of Science and Technology, Wuhan, China; ^2^ Department of Respiratory and Critical Care Medicine, Taikang Tongji (Wuhan) Hospital, Wuhan, China

**Keywords:** spleen tyrosine kinase, asthma, steroid resistance, airway inflammation, glucocorticoid receptor

## Abstract

Background: Regulation or restoration of therapeutic sensitivity to glucocorticoids is important in patients with steroid-resistant asthma. Spleen tyrosine kinase (Syk) is activated at high levels in asthma patients and mouse models, and small-molecule Syk inhibitors such as R406 show potent anti-inflammatory effects in the treatment of immune inflammatory diseases. Several downstream signaling molecules of Syk are involved in the glucocorticoid response, so we hypothesized that R406 could restore sensitivity to dexamethasone in severe steroid-resistant asthma.

**Objective:** To discover the role of the Syk inhibitor R406 in glucocorticoid resistance in severe asthma.

**Methods:** Steroid-resistant asthma models were induced by exposure of C57BL/6 mice to house dust mite (HDM) and β-glucan and by TNF-α administration to the bronchial epithelial cell line BEAS-2B. We evaluated the role of the Syk inhibitor R406 in dexamethasone (Dex)-insensitive airway inflammation. Pathological alterations and cytokines in the lung tissues and inflammatory cells in BALF were assessed. We examined the effects of Dex or R406 alone and in combination on the phosphorylation of MAPKs, glucocorticoid receptor (GR) and Syk, as well as the transactivation and transrepression induced by Dex in mouse lung tissues and BEAS-2B cells.

**Results:** Exposure to HDM and β-glucan induced steroid-resistant airway inflammation. The Syk inhibitor R406 plus Dex significantly reduced airway inflammation compared with Dex alone. Additionally, TNF-α-induced IL-8 production in BEAS-2B cells was not completely inhibited by Dex, while R406 markedly promoted the anti-inflammatory effect of Dex. Compared with Dex alone, R406 enhanced Dex-mediated inhibition of the phosphorylation of MAPKs and GR-Ser226 induced by allergens or TNF-α *in vivo* and *in vitro*. Moreover, R406 also restored the impaired expression and nuclear translocation of GRα induced by TNF-α. Then, the activation of NF-κB and decreased HDAC2 activity in the asthmatic model were further regulated by R406, as well as the expression of GILZ.

**Conclusions:** The Syk inhibitor R406 improves sensitivity to dexamethasone by modulating GR. This study provides a reference for the development of drugs to treat severe steroid-resistant asthma.

## Introduction

Bronchial asthma is characterized by airflow obstruction, airway hyperresponsiveness (AHR), airway remodeling and recurrent episodes and affects over 300 million people worldwide (Reddel et al., 2015; Bush, 2019; Roskoski, 2020). Inhaled corticosteroids (ICSs), also known as glucocorticoids (GCs), have been the cornerstone of asthma treatment for their efficacy in suppressing airway inflammation (Wadhwa et al., 2019). However, 5–10% of patients with severe steroid-resistant asthma still have unsatisfactory symptom control even with high-dose or systemic corticosteroid therapy plus controller medications (Hossen et al., 2015; Peters et al., 2019; Nabe, 2020).

Normally, GCs rapidly diffuse into airway tissue, where they bind and activate glucocorticoid receptor (GR; gene symbol NR3C1). GC-bound GR translocates from the cytoplasm into the nucleus and exerts anti-inflammatory effect in two ways: transrepression and transactivation (Hansbro et al., 2017). Transrepression refers to GR preventing the transcription of proinflammatory genes by reducing the activity of transcription factor complexes, such as nuclear factor (NF)-κB and the transcription corepressor histone deacetylase (HDAC)2. In contrast, GR interacts with glucocorticoid response elements (GREs), such as glucocorticoid-induced leucine zipper (GILZ) and glucocorticoid-induced transcript-1 (GLCCI1), to induce the expression of anti-inflammatory genes, which is called transactivation (Izuhara et al., 2014; Hansbro et al., 2017; Wadhwa et al., 2019; Palumbo et al., 2020). Allergens or inflammatory mediators induce the phosphorylation of mitogen-activated protein kinases (MAPKs), which leads to the phosphorylation of GR-Ser226 and reduces the responsiveness of asthma to GCs (Khorasanizadeh et al., 2017; Marchini et al., 2019). In patients with severe steroid-resistant asthma, the expression of tumor necrosis factor (TNF)-α in the lung was significantly increased and could not be inhibited by GCs (Hansbro et al., 2011; Long et al., 2020; Nabe, 2020). Airway epithelial cells or smooth muscle cells stimulated by TNF-α alone or in combination with other cytokines, such as interferon-γ, show steroid resistance (Chang et al., 2015; Chachi et al., 2017; Kobayashi et al., 2017). However, TNF-α and MAPK inhibitors are not suitable for asthma treatment due to numerous adverse effects associated with long-term use (Wenzel et al., 2009; Hansbro et al., 2011). Although progress has been made in the study of mechanisms, there are currently few safe and effective drugs to improve steroid resistance.

Spleen tyrosine kinase (Syk) is a nonreceptor cytoplasmic protein tyrosine kinase that is predominantly found in hematopoietic cells and is also expressed in nonimmune systems, including fibroblasts, liver cells and airway epithelial cells (Mocsai et al., 2010; Geahlen, 2014; Barnes, 2016). The signal switch Syk activates a variety of downstream signaling pathways and plays an essential role in regulating immune-inflammatory responses (Geahlen, 2014; Alhazmi, 2018). Small-molecule Syk inhibitors, such as R406, have been shown to be protective in the treatment of immune-inflammatory diseases, including allergic rhinitis, rheumatoid arthritis, IgA nephropathy, and acute lung injury (Wenzel et al., 2009; Weinblatt et al., 2010; Wu et al., 2019; Kost-Alimova et al., 2020). Most notably, increased expression or activity of Syk was reported in asthmatic airway inflammation (Patel et al., 2018). Syk inhibition significantly suppresses TNF-α-induced phosphorylation of MAPKs and p65 NF-κB, downregulating the levels of IL-6 and nitric oxide (Ashikawa et al., 2002; Takada and Aggarwal, 2004; Ulanova et al., 2005; Ulanova et al., 2006; Wang et al., 2006). In addition, a Syk inhibitor downregulated TNF-α-induced IL-8 expression in airway smooth muscle cells and human rhinovirus-induced IL-8 expression in the primary airway epithelial cells of asthmatic patients (Tabeling et al., 2017; Bleecker et al., 2020). It is well established that Syk inhibitors are highly effective in alleviating airway inflammation, but there has been no report about whether these inhibitors can regulate abnormal GC and GR signaling and thereby restore sensitivity to GCs.

In this study, we hypothesized that the Syk inhibitor R406 could restore steroid sensitivity in the asthmatic airway, and the molecular mechanism was elucidated.

## Materials and Methods

### Animals

Female C57BL/6 mice (6–8 weeks) were purchased from Beijing HFK Bioscience Co., Ltd. (Beijing, China), raised in a specific pathogen-free room in a controlled environment (24 ± 2°C, 40 ± 10% humidity, a 12 h light/dark cycle) and fed standard chow and filtered tap water ad libitum. All animal experiments met internationally accepted ethics standards and were approved by the Laboratory Animal Welfare and Ethics Committee of Tongji Hospital, Huazhong University of Science and Technology.

### HDM + Curdlan-Induced Steroid-Resistant Asthma and Treatment in Mice

Mice were randomly divided into six groups: control, R406, asthma model, asthma model + R406, asthma model + Dex treatment, and asthma model + Dex + R406 treatment. Mice in the model group were lightly anesthetized and intranasally instilled with a solution of lyophilized HDM extract (15 μg, Greer Lab, Lenoir, NC) and curdlan (20 μg, also known as β-glucan, Sigma–Aldrich, St. Louis, Mo) in 40 µl of saline three times/week for three consecutive weeks; analysis was performed on Day 20. Mice were intragastrically administered R406 (5 mg/kg, MCE, Shanghai, China) and/or Dex (1 mg/kg, MCE) by intraperitoneal injection from Days 16 to 19. Mice in the control group received the same volume of vehicle. R406 (C_28_H_29_FN_6_O_8_S) is an orally available and ATP-competitive inhibitor of Syk activity, the half-life of R406 could potentially allow once or twice-daily dosing (Baluom et al., 2013). The intake of 5 mg/kg/day of R406 could effectively inhibit inflammatory molecules in mice reported by previous research (Kitai et al., 2017).

### Cell Counting in Bronchoalveolar Lavage Fluid

Twenty-four hours after the last treatment, the mice were anesthetized with pentobarbital sodium (0.1%) and sacrificed. BALF was collected by cannulating the trachea and intratracheal instillation with 700 μl of saline in triplicate, followed by centrifugation at 1,000 rpm at 4°C. The pelleted cells were resuspended in 500 μl of PBS, and total white blood cells were determined using a hemocytometer. Macrophages, neutrophils, eosinophils and lymphocytes were counted after Liu’s staining according to the manufacturer’s protocol.

### Lung Histology

The left lungs were fixed in 4% formalin for 24 h before being embedded in paraffin. Lung sections (5 μM) from 5-7 mice each group were stained with hematoxylin and eosin (HE) or periodic acid-Schiff (PAS). Experienced pathologists performed the histopathological analyses in a blinded manner. For HE staining, eight-ten random fields for each lung section at ×200 magnification were selected, peribronchial inflammation was scored according to the following scale: 0, no cell infiltration; 1, few cells; 2, a ring of inflammatory cells 1 cell layer deep; 3, a ring of inflammatory cells 2–4 cells deep; and 4, a ring of inflammatory cells more than 4 cells deep (Park et al., 2020; Zhang et al., 2021). For PAS staining, the number of PAS-staining-positive cells was counted in also eight-ten random fields for each lung section, and the ratio of PAS-positive cells to epithelial cells in the airway was calculated (Zhang et al., 2021).

### Cell Culture, Stimulation, and Treatment

Human bronchial airway epithelial BEAS-2B cells were cultured until they reached confluence in Dulbecco’s modified Eagle’s medium (DMEM, Life Technologies, China) supplemented with 10% fetal bovine serum (Gibco, Life Technologies, United States) at 37 °C in humidified air with 5% CO_2_. BEAS-2B cells were plated into 6-well or 12-well plates coated with fibronectin (10 ng/ml, BD Bioscience, New Jersey, United States). Upon reaching confluence, R406 (1 μM) or DMSO (vehicle control, Sigma–Aldrich) was added to the medium and incubated for 30 min. Then, BEAS-2B cells were incubated with Dex (1 μΜ) for 2 h following treatment with or without TNF-α (10 ng/ml, PeproTech, Rocky Hill, United States). At the indicated times, protein phosphorylation levels were measured by Western blotting. Gene expression and cytokine release were measured using PCR and ELISA 24 h after stimulation.

### RNA Extraction, Reverse Transcription, and Quantitative PCR

The lung tissues and cells cultured *in vitro* were lysed, total RNA was prepared using TRIzol reagent (Takara, Otsu, Japan). RNA concentration and purity were measured by a NanoDrop 2000 (Thermo Scientific). cDNA was synthesized using 500 ng of total RNA, and qPCR was performed according to the instructions of the SYBR Green RT kit (Takara) in a real-time PCR instrument (Bio–Rad, IT-IS, Ireland). The amplification conditions were 95°C for 5 min, followed by 40 cycles of 95°C for 10 s, 60°C for 10 s, and 72°C for 10 s. Reactions were followed by a melt analysis (95°C for 15 s, 60°C for 20 s, 95°C for 15 s with ramping to 95°C over 20 min) to confirm primer specificity. The relative expression of targeted genes was normalized against the expression of glyceraldehyde 3-phosphate dehydrogenase (GAPDH) and calculated using the 2^−ΔΔCt^ comparative method. The sequences of the primers used were list as follows (Table 1).

**TABLE 1:** List of PCR primers used in this study.

Gene name		Sequence (5′ to 3′)
Human		
GAPDH	F	CCT​GAC​CTG​CCG​TCT​AGA​AA
	R	CTCCGACGCCTGCTTCAC
IL-8	F	TCT​GGC​AAC​CCT​AGT​CTG​CT
	R	AAA​CCA​AGG​CAC​AGT​GGA​ACG​C
GILZ	F	GGC​CAT​AGA​CAA​CAA​GAT​CG
	R	ACT​TAC​ACC​GCA​GAA​CCA​CCA
GLCCI1	F	GGG​AAG​GAA​GAA​GTA​TCC​AAG​C
	R	GCG​AGT​ACT​ACT​GCT​CCG​GTA
Mouse		
GAPDH	F	TGG​CCT​TCC​GTG​TTC​CTA​C
	R	GAG​TTG​CTG​TTG​AAG​TCG​CA
IL-5	F	GCA​ATG​AGA​CGA​TGA​GGC​TTC
	R	GCC​CCT​GAA​AGA​TTT​CTC​CAA​TG
IL-13	F	CAG​CCT​CCC​CGA​TAC​CAA​AAT
	R	GCG​AAA​CAG​TTG​CTT​TGT​GTA​G
IL-17F	F	TGC​TAC​TGT​TGA​TGT​TGG​GAC
	R	CAG​AAA​TGC​CCT​GGT​TTT​GGT
TNF-α	F	CAG​GCG​GTG​CCT​ATG​TCT​C
	R	CGA​TCA​CCC​CGA​AGT​TCA​GTA​G

### Enzyme-Linked Immunosorbent Assay

Epithelial BEAS-2B cells were pretreated with R406 for 30 min, followed by exposure to TNF-α (10 ng/ml) for 24 h in the presence/absence of dexamethasone (1 µM) for 2 h. The concentrations of IL-8 (CXCL8) in the cell culture supernatants were examined using a commercially available enzyme-linked immunosorbent assay kit (DY208, R&D Systems, Minneapolis, MN) according to the manufacturer’s instructions.

### Western Blot Analysis

Lung tissues or cell lysates were prepared according to standard procedures. Briefly, total proteins were extracted using RIPA lysis buffer (Biouniquer, United States) supplemented with phosphatase inhibitors and a protease inhibitor cocktail (MCE). Cytoplasmic and nuclear proteins were extracted from cells using a nuclear extraction kit. After being quantified, the total protein was adjusted with 5× loading buffer, and the samples were boiled for 8 min at 100°C. Equal amounts of protein were separated by 10–12% sodium dodecyl sulfate (SDS) polyacrylamide gels and transferred onto polyvinylidene difluoride membranes (Thermo Scientific^TM^, Rockford, United States), which were then blocked with 5% skim milk in TBST (TBS + 0.05% Tween-20) at room temperature for 1 h and then washed with TBST three times. The membranes were incubated with the indicated primary antibodies against GRα (Abcam, UK), phospho-Syk (Tyr323) (Affinity Biosciences, Jiangsu, China), phospho-GR (Ser226), phospho-p38 MAPK (Thr180/Tyr182), phospho-ERK1/2 (Thr202/Tyr204), phospho-JNK (Thr183/Tyr185), phospho-p65 NF-κB (Ser536), HDAC2, GAPDH, and β-Actin (Cell Signaling Technology, Danvers, MA) separately overnight at 4°C. Afterward, the membranes were incubated with anti-rabbit or anti-mouse HRP-conjugated secondary antibodies (Aspen Biological, Wuhan, China) at room temperature for 1 h. Digital images were subsequently captured with a Chemical Imaging System (BioRad, Hercules, CA) and quantified by ImageJ software (NIH, Littleton, CO).

### Immunofluorescence Analysis

For immunofluorescence analysis, BEAS-2B cells (1 × 10^4^ cells) were seeded on fibronectin-coated coverslips in 6-well plates. To evaluate the effect of R406 on the nuclear translocation of GRα, cells were pretreated for 30 min with 1 μM R406 and thereafter activated with Dex in the presence or absence of TNF-α. After 12 h, the cells were washed three times with PBS (137 mm NaCl, 2.7 mm KCl, 10 mm Na_2_HPO_4_, 1.8 mm KH_2_PO_4_) and fixed with 4% paraformaldehyde for 15 min. The cells were permeabilized with 0.5% Triton X-100 (Solarbio, Beijing, China) for 15 min and blocked with 10% goat serum (Aspen Biological, Wuhan, China) in PBS for 30 min at room temperature. The coverslips were then incubated with rabbit anti-GRα (1:100) overnight at 4°C. After three washes with PBS, GRα was labeled with the FITC-conjugated goat anti-rabbit IgG (H + L) secondary fluorescent antibody (1:100, Servicebio, Wuhan, China) for 1 h. Cell nuclei were stained with 4′,6-diamidino-2-phenylindole (DAPI, Aspen Biological, Wuhan, China). Images were photographed by an Olympus BX51 fluorescence microscope (Olympus Corporation, Tokyo, Japan) and processed by ImageJ software.

### Statistical Analysis

Statistical analysis was performed with GraphPad Prism version 7.0 (GraphPad Software, San Diego, CA). All results are presented as the mean ± SEM. Student’s t test was used to compare two groups. One-way ANOVA followed by Tukey’s post-*hoc* multiple comparison tests were used for analysis among multiple groups. Each experiment was performed at least in triplicate as indicated; a *p* value < 0.05 was considered statistically significant.

## Results

### The Syk Inhibitor R406 Enhances the Inhibitory Effect of Dexamethasone on Lung Inflammation in Asthmatic Mice

**FIGURE 1:**
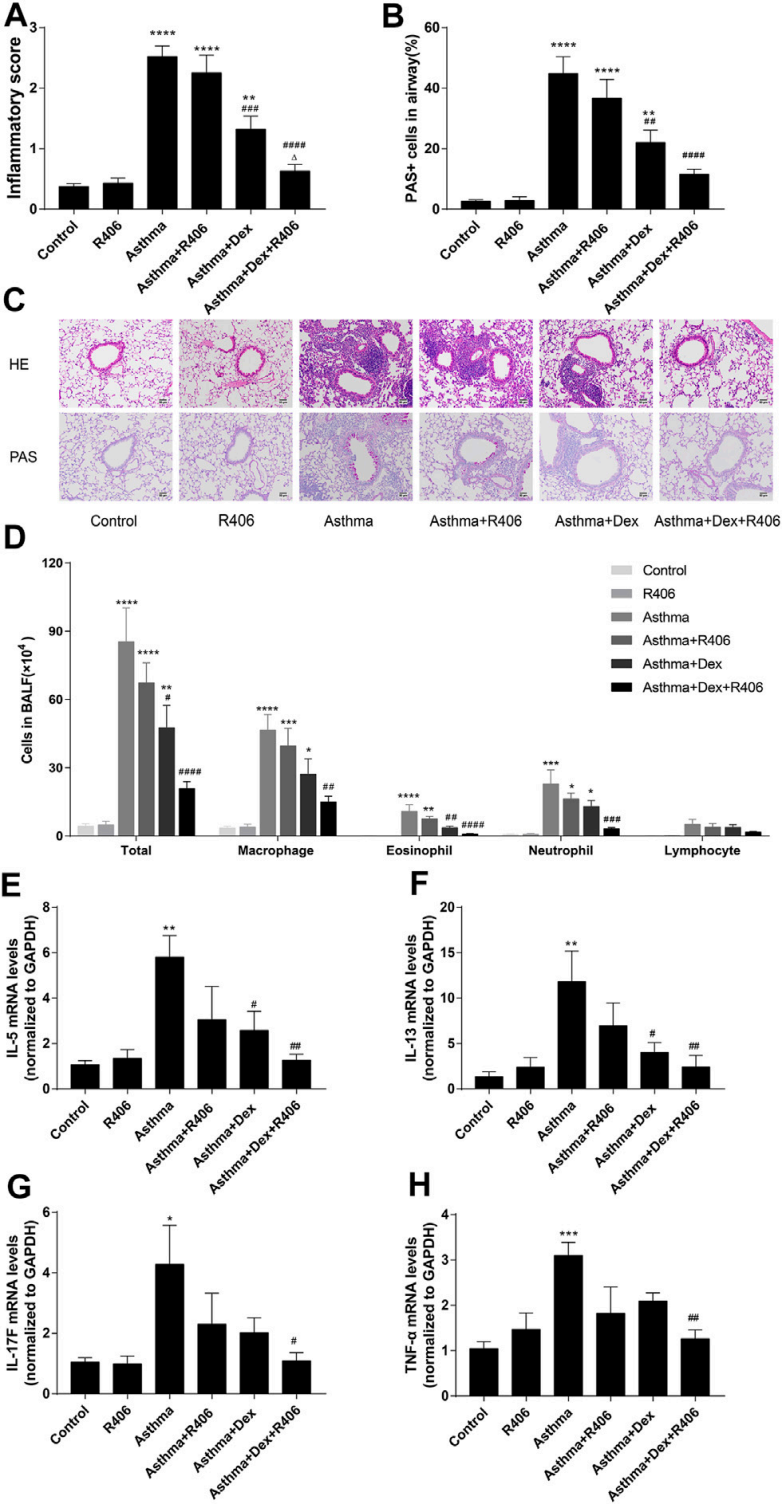
R406 enhances the inhibitory effect of dexamethasone on lung inflammation in asthmatic mice. **(A,B)** The allergens HDM (15 μg) and β-glucan (20 μg) in 40 μl of saline three times/week for three consecutive weeks were used to induce asthma in mice, and lung histology was assessed 24 h after the last challenge as described in the Methods. Lung inflammation scores were estimated from HE staining and PAS-positive cells per total epithelial cells in the airway. **(C)** Representative HE-stained and PAS-stained lung tissue sections (original magnification ×200, scale bar = 50 μM). **(D)** Total cell, macrophage, eosinophil, neutrophil, and lymphocyte counts in BALF. **(E–H)** Cytokine expressions in lung issues were detected by PCR. *n* = 5–7 mice per group from two independent experiments. *p < 0.05, **p < 0.01, ***p < 0.001, ****p < 0.0001 compared with the control group; ^#^ *p* < 0.05, ^##^ *p* < 0.01, ^###^ *p* < 0.001, ^####^ *p* < 0.0001 compared with the asthma model group. ^△^ *p* < 0.05, compared with the asthma model + Dex treatment group.

The effects of R406 and Dex on airway inflammation of the lung tissues were assessed by HE staining. A significant increase in inflammatory cell infiltration was observed around the airway in asthmatic mice and was slightly reduced by Dex treatment alone, which was a more robust effect than that in control mice. Compared with Dex alone, treatment with R406 plus Dex significantly reduced peritracheal infiltration of inflammatory cells, and there was no significant difference from that in the control group (Figures 1A,C). PAS staining (Figures 1B,C) showed similar effects on airway mucus production.

Consistent with the histologic appearance, the numbers of total cells, macrophages, neutrophils, and eosinophils in BALF were significantly increased in HDM- and curdlan-sensitized mice (*p* < 0.0001, *p* < 0.0001, *p* < 0.001, *p* < 0.001, respectively, Figure 1D). Dex treatment markedly decreased the numbers of total white blood cells (*p* < 0.05) and eosinophils (*p* < 0.01) but had little effect on neutrophils. However, a significant reduction in neutrophil numbers was observed in the Dex plus R406 group (*p* < 0.0001). Compared with that in response to Dex alone, the numbers of total cells (*p* < 0.0001), macrophages (*p* < 0.01), eosinophils (*p* < 0.0001), and neutrophils (*p* < 0.001) decreased significantly after oral administration of R406, and there was no more significant difference compared with that in the control group. Lymphocytes showed a similar trend, but there was no significant difference. Importantly, there was little difference between control and R406 mice that were not stimulated with the allergen. We then examined the effect of R406 and Dex on the lung generation of cytokines. We found that the expression of IL-5 (Figure 1E, *p* < 0.01), IL-13 (Figure 1F, *p* < 0.01), IL-17F (Figure 1G, *p* < 0.05), and TNF-α (Figure 1H, *p* < 0.001) were increased in the lung tissue in asthmatic mice as compared with those of the control groups. Dex treatment alone markedly decreased the expression of IL-5 (*p* < 0.05) and IL-13 (*p* < 0.05) but failed to alter the levels of IL-17F and TNF-α, which were significantly reduced by Dex combined with R406.

### R406 Restores the Suppressive Effect of Dexamethasone on TNF-α-Induced IL-8 Production in Airway Epithelial Cells

**FIGURE 2:**
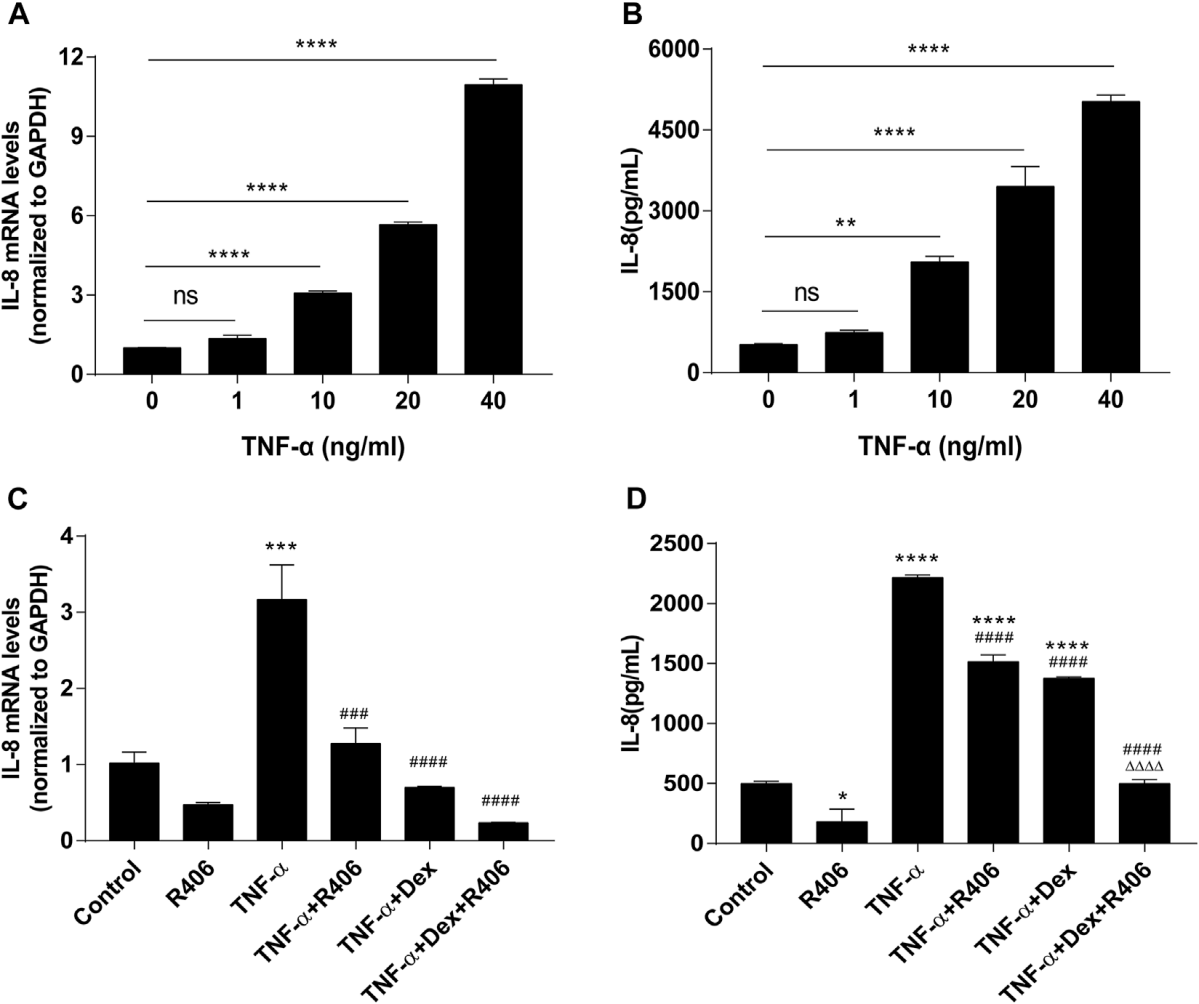
The Syk inhibitor R406 promotes the inhibitory effect of dexamethasone on TNF-α-induced IL-8 in airway epithelial cells. **(A,B)** BEAS-2B cells were stimulated with different concentrations (0, 1, 10, 20, 40 ng/ml) of TNF-α for 24 h, and the mRNA expression levels of IL-8 were measured by PCR **(A)** and ELISA **(B)**. *n* = 3, ** *p* < 0.01, **** *p* < 0.0001, ns indicates no significant difference. **(C,D)** BEAS-2B cells were pretreated with R406 (1 μM) for 30 min, incubated with Dex (1 μM) for 2 h, and stimulated with TNF-α (10 ng/ml) for 24 h. The expression of IL-8 was measured by PCR **(C)** and ELISA **(D)**. *n* = 3/group, *p < 0.05, ***p < 0.001, compared with the control group; ^###^ *p* < 0.001, ^####^ *p* < 0.0001, compared with the TNF-α group; ^△△△△^ *p* < 0.0001, compared with the TNF-α +Dex group.

TNF-α upregulated the expression of IL-8 in BEAS-2B cells in a dose-dependent manner, as shown by PCR (Figure 2A) and ELISA (Figure 2B). The expression of IL-8 was significantly higher than that in the control group when the concentration of TNF-α was 10 ng/ml (*p* < 0.0001). Therefore, 10 ng/ml was selected as the stimulation concentration in the follow-up experiments. First, we investigated the expression of IL-8 in BEAS-2B cells after pretreatment with dexamethasone alone (Dex, 1 μM) or R406 (1 μM). Similar to what was observed in the asthmatic model, Dex inhibited TNF-α-mediated induction of IL-8 (38%), but the expression of IL-8 was still significantly higher than that in the control group (*p* < 0.0001), showing insensitivity to Dex in BEAS-2B cells (Figure 2D). However, 30 min after incubation with R406, Dex-mediated inhibition of TNF-α-induced IL-8 reached 78%, and at this time, IL-8 decreased to the level observed in control group, and there was a significant difference between the two groups (*p* < 0.0001). Similar results were shown for IL-8 mRNA levels, although Dex significantly reduced IL-8 expression, and R406 enhanced the inhibitory effect (*p* < 0.0001, Figure 2C). These data suggest that TNF-α attenuates the anti-inflammatory effect of Dex, while the Syk inhibitor R406 restores Dex sensitivity.

### R406 Restores Steroid Sensitivity and Suppresses Mitogen-Activated Protein Kinases and GR-Ser226 Phosphorylation

**FIGURE 3:**
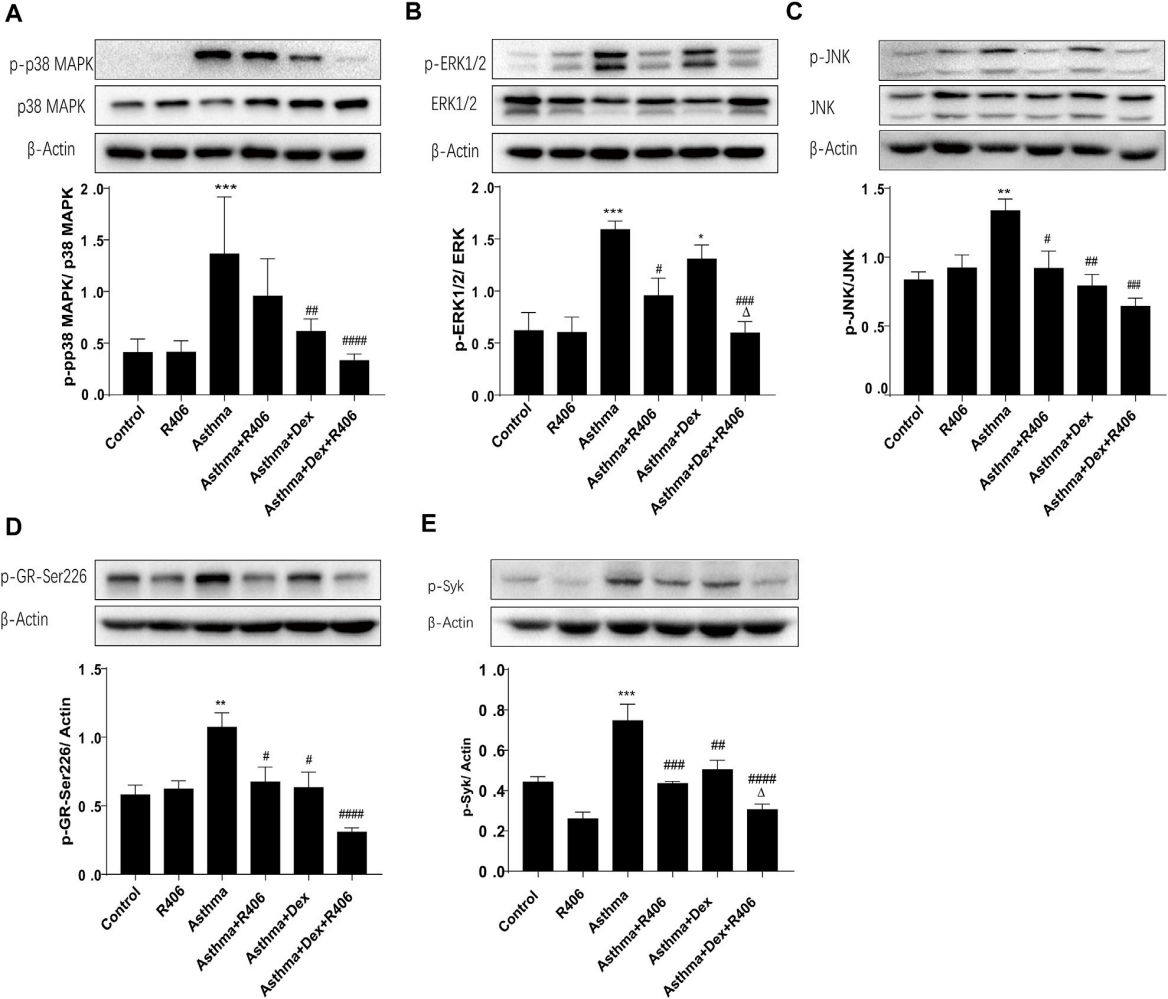
The Syk inhibitor R406 enhances Dex-mediated inhibition of MAPK and Syk phosphorylation in an asthmatic mouse model. Twenty-four hours after the last challenge, the phosphorylation of p38 MAPK **(A)**, ERK1/2 **(B)**, JNK **(C)**, GR-Ser226 **(D)** and Syk **(E)** was quantified in the right lungs by Western blotting. *n* = 5/group, *p < 0.05, **p < 0.01, ***p < 0.001, compared with the control group; ^#^ *p* < 0.05, ^##^ *p* < 0.01, ^###^ *p* < 0.001, ^####^ *p* < 0.0001, compared with the asthma model group. ^△^ *p* < 0.05, compared with the asthma model + Dex treatment group.

Activated MAPKs that phosphorylate and deactivate the GR are believed to be essential in steroid resistance. In the lung tissues of asthmatic mice, Western blotting revealed significantly increased phosphorylation levels of p38 MAPK (Figure 3A), ERK1/2 (Figure 3B), and JNK (Figure 3C). However, the phosphorylation of MAPKs was significantly inhibited by R406 combined with Dex (*p* < 0.001, *p* < 0.01, and *p* < 0.01, respectively). As R406 inhibited the phosphorylation of MAPKs, we then wanted to characterize the effects of R406 on the phosphorylation of GR-Ser226, which is associated with a reduction in GR function. As shown in Figure 3D, GR-Ser226 showed a similar trend as MAPKs; the phosphorylation of GR-Ser226 was increased in the lung tissues of asthmatic mice (*p* < 0.05), and these levels could be restored to normal by Dex and R406 treatment. In the meantime, we detected the phosphorylation of Syk (Figure 3E). Similar to the pattern of activated MAPKs and GR, the phosphorylation of Syk in lung tissues of asthma group was markedly enhanced and could be impaired by R406 or Dex, and even more suppressed by Dex combining with R406 (*p* < 0.05).

**FIGURE 4:**
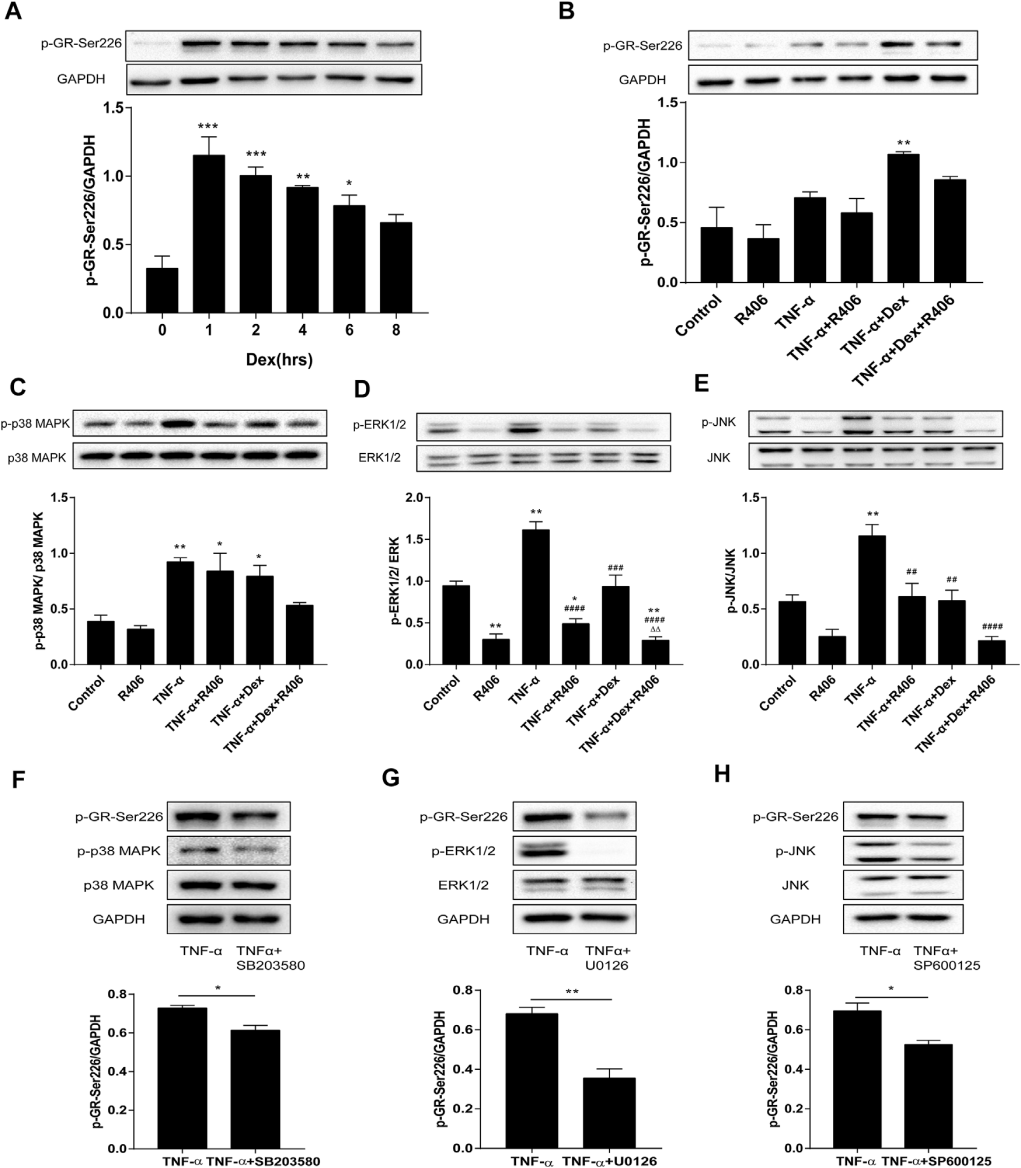
The Syk inhibitor R406 impairs the phosphorylation of GR-Ser226 by inhibiting MAPK activation. **(A)** BEAS-2B cells were treated with Dex (1 μM) for 1, 2, 4, 6, and 8 h, and the phosphorylated protein levels of GR-Ser226 were measured by Western blotting. *n* = 3/group, * *p* < 0.05, ** *p* < 0.01, *** *p* < 0.001. **(B–E)** BEAS-2B cells were pretreated with R406 (1 M) for 30 min, incubated with Dex (1 μM) for 2 h, and stimulated with TNF-α (10 ng/ml) for 1 h or 20 min. The phosphorylation levels of GR-Ser226 **(B)**, p38 MAPK **(C)**, ERK 1/2 **(D)** and JNK **(E)** were measured by Western blotting. *n* = 3/group, * *p* < 0.05, ** *p* < 0.01, compared with the control group; ^##^ *p* < 0.01, ^####^ *p* < 0.0001, compared with the TNF-α group; ^△△^ *p* < 0.01, compared with the TNF-α +Dex group. **(F–H)** BEAS-2B cells were pretreated with a p38MAPK inhibitor (SB203580,10 μM), ERK1/2 inhibitor (U0126,10 μM) and JNK inhibitor (SP600125, 5 μM) for 30 min and stimulated with TNF-α (10 ng/ml) for 1 h, followed by Western blotting to measure the phosphorylation levels of p38MAPK, ERK1/2, JNK and GR-Ser226. *n* = 3/group, *p < 0.05, **p < 0.01.

Glucocorticoids decreased GR expression and phosphorylation in a time-dependent manner. BEAS-2B cells were treated with Dex, and GR phosphorylation at the Ser226 residue reached a peak after 1 h and decreased gradually with the time until 8 h (Figure 4A). Therefore, the phosphorylation of GR-Ser226 was more pronounced with the addition of Dex than with stimulation by TNF-α alone but decreased after pretreatment with R406 (*p* < 0.01, Figure 4B). Consistent with the *in vivo* results, cells treated with TNF-α for 20 min showed significant activation of MAPKs (Figures 4C–E). Further analysis showed that the phosphorylation levels of MAPKs could be partially attenuated by Dex, and this suppression was amplified when Dex was combined with R406. Compared with the effect of Dex alone, the phosphorylation levels of p38 MAPK, ERK1/2 and JNK induced by TNF-α decreased by 33, 75, and 63%, respectively, after R406 pretreatment.

Next, we used inhibitors of MAPKs in BEAS-2B cells to study the effects of MAPK phosphorylation on GR-Ser226. As expected, the phosphorylation of GR-Ser226 was reduced after pretreatment with a p38 MAPK antagonist (SB203580, Figure 4F, *p* < 0.05) compared with TNF-α stimulation, and similar effects were observed with specific antagonists of ERK1/2 (U0126, Figure 4G, *p* < 0.01) and JNK (SP600125, Figure 4H, *p* < 0.05). These results indicated that MAPK activation played a role in GR-Ser226 phosphorylation and that R406 could inhibit the phosphorylation of GR-Ser226 by inhibiting the MAPK signaling pathway.

### R406 Improves TNF-α-Induced GRα Nuclear Translocation Without Affecting the GRβ/Grα Ratio

**FIGURE 5:**
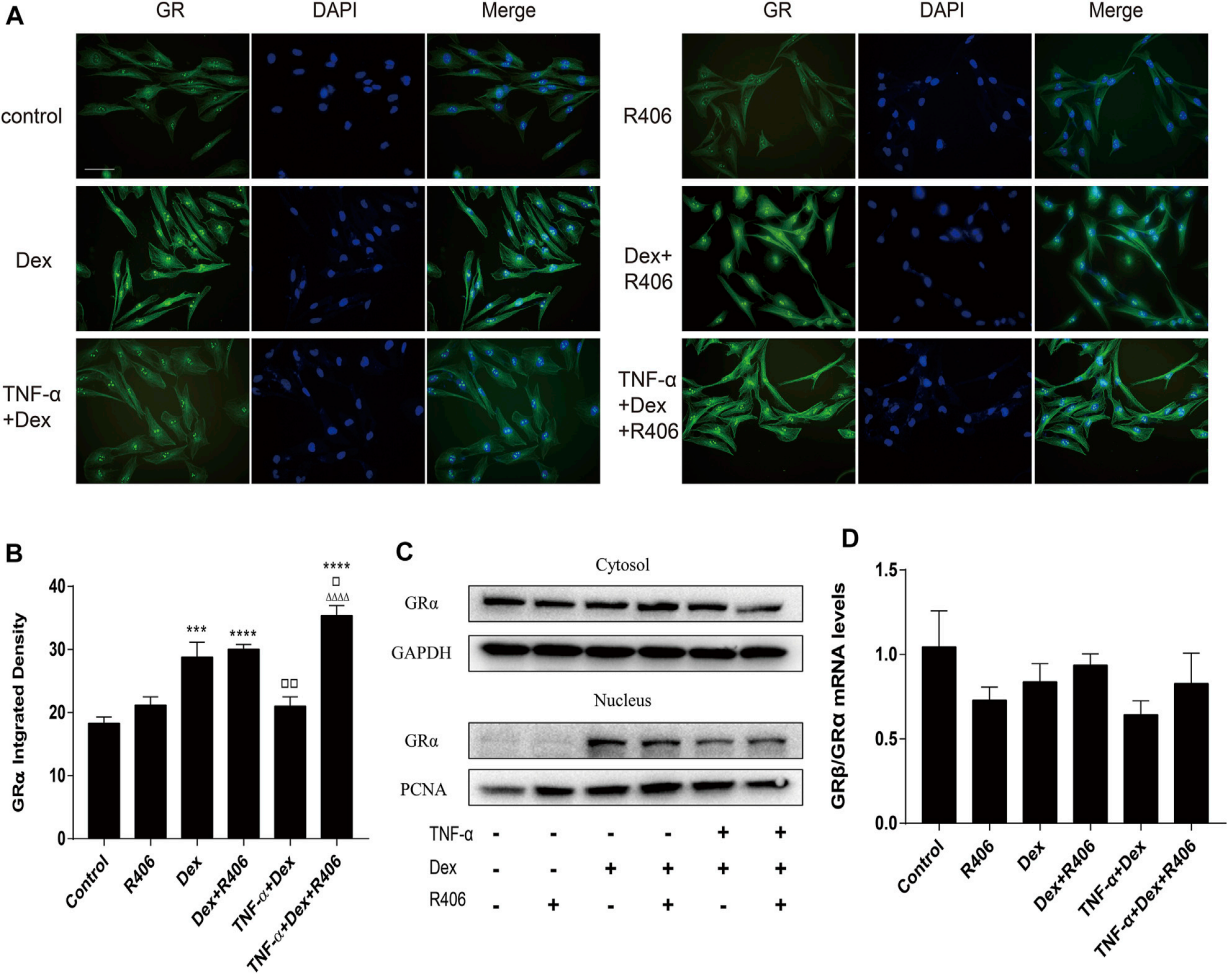
The Syk inhibitor R406 improves TNF-α-induced GRα nuclear translocation without affecting the GRβ/GRα ratio. **(A–C)** BEAS-2B cells were pretreated with R406 (1 μM) for 30 min and incubated with Dex (1 μM) or with Dex (1 μM) plus TNF-α (10 ng/ml) for 12 h. GRα was dyed green, and the cell nuclei were stained with DAPI (blue). The expression and distribution of GRα in cells were analyzed by immunofluorescence **(A,B)** and Western blotting **(C)**. **(D)** The mRNA levels of GRβ and GRα relative to GAPDH were measured by qPCR. *n* = 3/group, Scale bar = 50 μM; ***p < 0.001, **p < 0.0001, compared with the control group; ▫*p* < 0.05, ▫▫*p* < 0.01, compared with the Dex group; ^△△△△^ *p* < 0.0001, compared with the TNF-α +Dex group.

There are three subtypes of glucocorticoid receptors, and GRα enters the nucleus after binding with the ligand. Therefore, we used immunofluorescence analysis to observe the distribution of GRα in the cytoplasm and nucleus after 12 h of Dex treatment. As shown in Figures 5A,B, GRα expression in the nucleus in the absence of Dex treatment was weak. However, Dex induced GRα expression and nuclear translocation in BEAS-2B cells, and TNF-α inhibited Dex-mediated GRα nuclear translocation. However, pretreatment with R406 restored TNF-α-mediated inhibition of Dex-dependent GRα nuclear translocation (Figure 5C). Some studies suggested that the ratio of GRβ/GRα was altered in steroid-resistant asthma, but this effect was not found in this study (Figure 5D).

### R406 Suppresses Inflammation by Regulating Dex-Induced Transrepression and Transactivation

**FIGURE 6:**
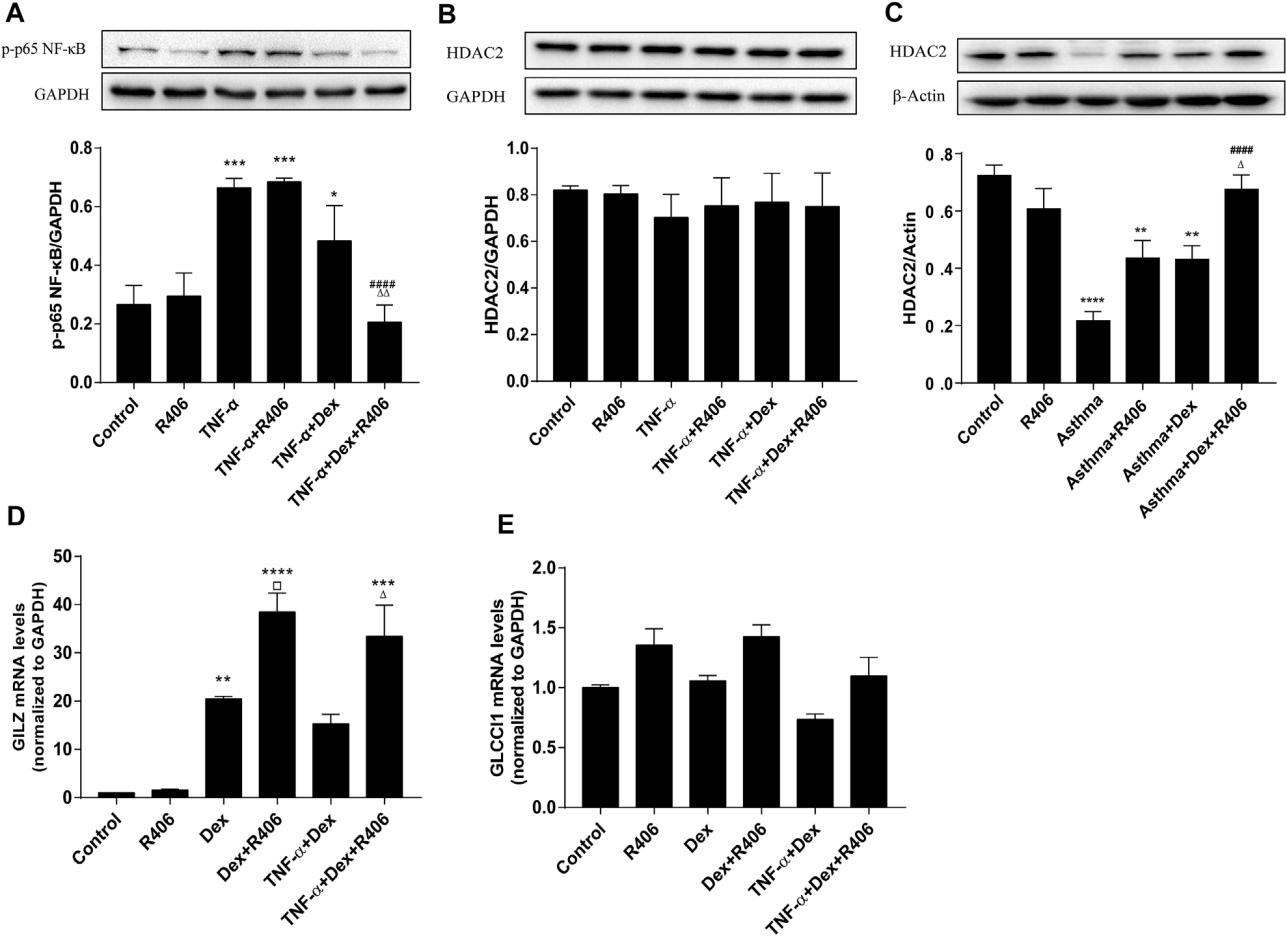
The Syk inhibitor R406 enhances the inhibitory effect of dexamethasone on transrepression and transactivation. **(A)** BEAS-2B cells were pretreated with R406 (1 μM) for 30 min, incubated with or without Dex (1 μM) for 2 h, and stimulated with TNF-α (10 ng/ml) for 10 min **(B)** BEAS-2B cells were pretreated with R406 (1 μM) for 30 min and incubated with TNF-α (10 ng/ml) or with Dex (1 μM) and TNF-α for 12 h. **(C)** The expression of HDAC2 in asthmatic mouse lung tissues was determined by Western blotting. *n* = 5/group. **(D,E)** BEAS-2B cells were pretreated with R406 (1 μM) for 30 min, incubated with Dex (1 μM) for 2 h and stimulated with or without TNF-α (10 ng/ml) for 24 h, and the mRNA levels of GILZ and CLCCI1 were measured by PCR. *n* = 3/group, *p < 0.05, **p < 0.01, ***p < 0.001, ****p < 0.0001, compared with the control group; ^##^ *p* < 0.01, ^####^ *p* < 0.0001, compared with the TNF-α or asthma model group; ▫*p* < 0.05 compared with the Dex group; ^△^ *p* < 0.05, ^△△^ *p* < 0.01, compared with the TNF-α +Dex group or asthma + Dex treatment group.

Glucocorticoids play anti-inflammatory roles through transrepression and transactivation after they translocate to the cell nucleus. Having found that R406 modulates the nuclear translocation induced by Dex, we examined whether R406 affected the expression of NF-κB, GILZ and GLCCI1. As shown in Figure 6A, the phosphorylation level of p65 NF-κB after TNF-α stimulation was more than twice that in the control group. Highly activated p65 NF-κB was not significantly affected by Dex treatment alone but was strikingly suppressed by pretreatment with R406 (*p* < 0.0001). NF-κB is a crucial regulator of HDAC2, a key protein in corticosteroid insensitivity. Surprisingly, we did not find any changes in HDAC2 in BEAS-2B cells in response to TNF-α stimulation for 24 h or Dex intervention (Figure 6B). Therefore, western blotting was used to measure HDAC2 expression in mouse lung tissues (Figure 6C). The expression of HDAC2 was significantly reduced in the asthma model group (*p* < 0.0001), was downregulated in Dex-treated mice (*p* < 0.01) and was restored by Dex and further potentiated by R406 treatment (*p* < 0.0001). Therefore, more HDAC2 expression was recovered in the Dex and R406 treatment groups than in the Dex group.

For transactivation induced by glucocorticoids, the PCR results showed that the increased expression of GILZ was dependent on Dex (*p* < 0.01). Although TNF-α had no significant effect on the hyperexpression of GILZ induced by Dex, R406 markedly enhanced the induction of GILZ (*p* < 0.05, Figure 6D). However, the expression of GLCCI1 was relatively stable; although there was no significant difference, R406 also tended to promote the expression of GLCCI1 (Figure 6E).

## Discussion

**FIGURE 7:**
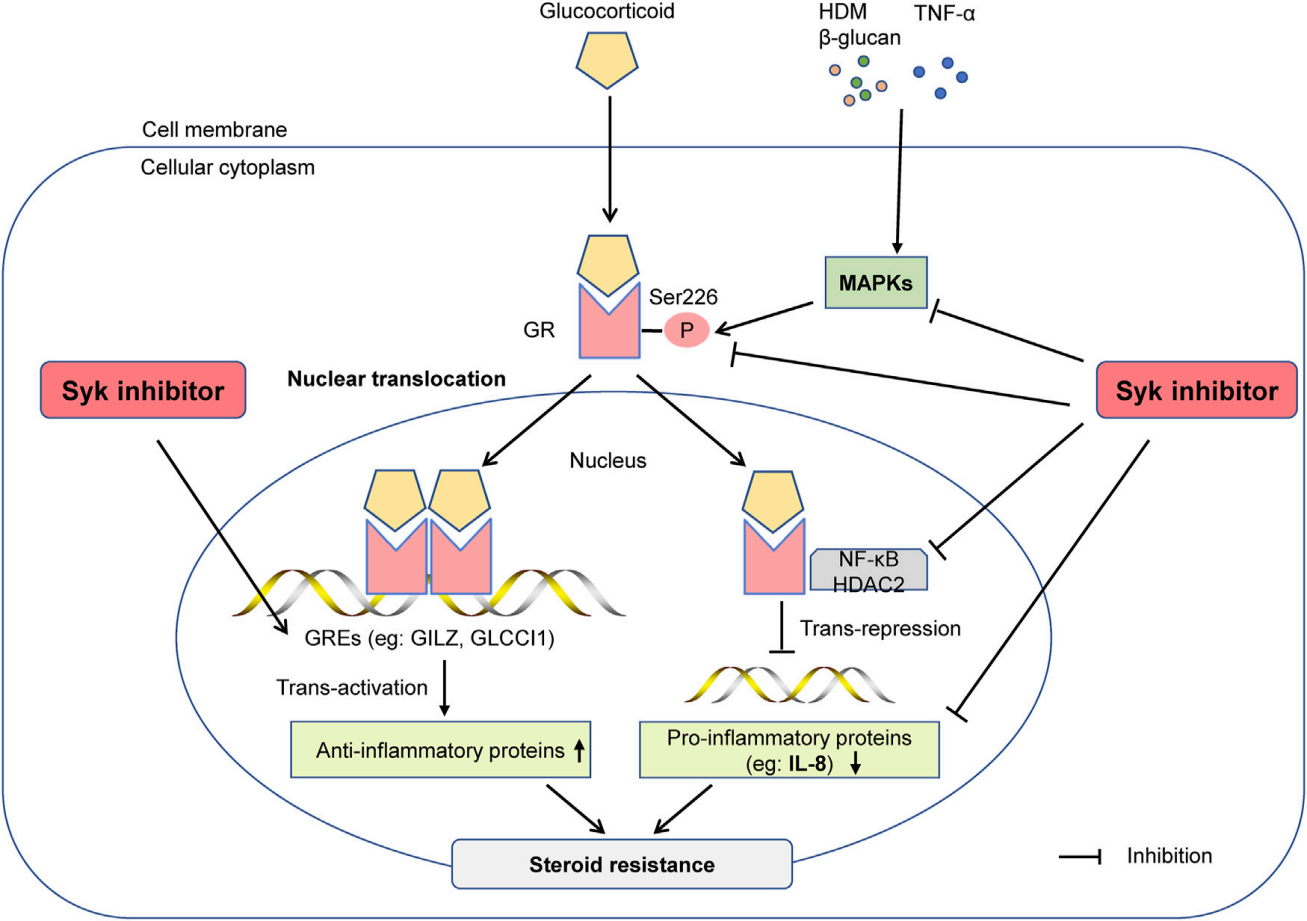
The Syk inhibitor R406 ameliorates glucocorticoid resistance in steroid-resistant asthma. Syk is essential for regulating inflammatory signaling in steroid-resistant asthma. The Syk inhibitor R406 can reduce the phosphorylation of GR-Ser226 by inhibiting allergen- or TNF-α-induced MAPK phosphorylation and restore the nuclear translocation of GR. R406 promotes dexamethasone-mediated inhibition of n NF-κB, restores HDAC2 activity, increases the expression of the glucocorticoid-responsive element GILZ, and reduces the expression of cytokines such as IL-8 to improve sensitivity to glucocorticoids in severe asthma.

This study showed that Syk was involved in steroid-resistant airway inflammation in asthma, and R406, a potent inhibitor of Syk, could improve glucocorticoid sensitivity in asthmatic mice *in vivo* and airway epithelial cells *in vitro*. Mechanistically, inhibition of Syk can restore the anti-inflammatory effect of glucocorticoids by reducing the phosphorylation of MAPKs and GR-Ser226, thereby improving the nuclear translocation of GRα and the transactivation and transrepression of glucocorticoids (Figure 7).

The role of environmental factors in the pathogenesis of asthma should not be ignored, which may worsen asthma symptoms. Some severe asthmatic patients have to receive increasing doses or systemic corticosteroids, which are associated with risks of developing adverse events, including but not limited to infections, cardiovascular disease, osteoporosis and metabolic disorders (Bleecker et al., 2020). Thus, it is absolutely imperative to elucidate the pathophysiology of glucocorticoid sensitivity and, if possible, look for potential drugs to improve steroid resistance. Zhang and other scholars found that the natural allergens HDM, and β-glucan, the main component of fungal cells, could induce severe steroid-resistant inflammation in mice (Hadebe et al., 2016; Zhang et al., 2017). In this study, in the presence of HDM and curdlan, inflammatory cells infiltrated the airways of mice, and goblet cells showed significant metaplasia; this effect was not inhibited by dexamethasone. Cell counts in BALF were analyzed, and Dex inhibited the increases in macrophages, eosinophils and lymphocytes induced by HDM and curdlan but had no significant effect on the number of neutrophils. Moreover, the expression of cytokines indicating an GC-sensitive, eosinophilic inflammatory such as IL-5 and IL-13 were responsive to Dex, while the expression of TNF-α and IL-17F, more biased towards a GC-resistant, neutrophilic inflammation, was less responsive to Dex treatment (Hansbro et al., 2011; Palumbo et al., 2020). Dex combined with R406 could significantly inhibit neutrophil infiltration and the expression of TNF-α and IL-17F. In addition, TNF-α-induced airway epithelial cells to secrete IL-8, a potent mediator that recruits neutrophils (Long et al., 2020; Nabe, 2020), which showed reduced sensitivity to dexamethasone *in vitro*. Compared with that in the mild-to-moderate asthma and healthy control groups, the level of airway IL-8 in patients with steroid-resistant asthma was elevated significantly (Pan et al., 2019). While the ability of Dex to inhibit TNF-α-induced IL-8 levels reflects sensitivity to glucocorticoids, Dex alone failed to alleviate airway inflammation, that is, usually steroid-resistant or refractory (Chachi et al., 2017; Kobayashi et al., 2017). Mercado et al found that in the peripheral blood mononuclear cells of patients with severe asthma, concentration of Dex that partly inhibited TNF-α-induced IL-8 was approximately 10 times higher than that in healthy controls or patients with mild asthma (Mercado et al., 2011). Similarily, in epithelial cells Dex alone inhibited TNF-α-mediated induction of IL-8, but the expression of IL-8 was still significantly higher than that in the control group. These results support previous findings that eosinophilic inflammation in asthmatic airways is steroid-sensitive, while neutrophilic inflammation is generally less sensitive (Nabe, 2020).

Syk, which is a crucial tyrosine kinase required for the expression of several proinflammatory cytokines, can be activated in the asthmatic airway, especially in epithelial cells (Penton et al., 2013; Yi et al., 2014; Patel et al., 2018). In our study, allergens HDM and curdlan induced a markedly phosphorylation of Syk, which could be inhibited by Dex combining R406. Airway inflammatory cell infiltration, mucus hypersecretion, and the expression of inflammatory factors, which were not or partly inhibited by Dex or R406 alone, were significantly improved by the combination of Dex and R406. The insensitivity of TNF-α-induced airway epithelial cells to dexamethasone could also be markedly restored by R406. Collectively, these results suggested a mechanism by which Syk affects asthmatic steroid-resistant inflammation, and the combination of R406 plus dexamethasone suppressed the inflammatory reaction in a synergistic manner.

As a signal switch, Syk regulates extracellular to intracellular transmission. The activation of Syk is important in initiating various inflammatory signaling pathways, including the MAPK pathway (Barnes, 2016; Coates et al., 2021). It is well established that Syk inhibitors can significantly inhibit MAPK activation. In addition, many studies have shown that the phosphorylation of GR at Ser226 following MAPK phosphorylation induced by TNF-α and other proinflammatory cytokines blocks GC binding and GR nuclear translocation, leading to steroid resistance (Bouazza et al., 2012; Kobayashi et al., 2017; Liu et al., 2018; Palumbo et al., 2020). The phosphorylation of GR-Ser226 in airway cells and peripheral blood mononuclear cells in severe asthma patients was higher than that in healthy controls or patients with mild-to-moderate asthma (Chang et al., 2015; Zhang et al., 2019). These results suggest that phosphorylated MAPKs are upregulated in lung tissue induced by HDM and curdlan, as well as BEAS-2B cells after TNF-α stimulation *in vitro*. To understand the effect of MAPK activation on the phosphorylation of GR-Ser226, BEAS-2B cells were incubated with specific p38 MAPK, ERK1/2 and JNK inhibitors. Consistent with previous reports, the phosphorylation of GR-Ser226 was attenuated accordingly. Moreover, the phosphorylation of GR-Ser226 was induced by Dex but decreased gradually with time, indicating the endogenous regulatory effect of steroids (Bouazza et al., 2012). TNF-α alone or in combination with Dex did not significantly change the phosphorylation level of GR-Ser226, similar to a previous study (Bouazza et al., 2012). Compared with those in the asthma model groups, similar to the activation of Syk, the phosphorylation levels of MAPKs and GR-Ser226 after Dex and R406 treatment were mitigated, which revealed that Syk may regulate the phosphorylation level of GR-Ser226 by mediating the activation of MAPKs, thus affecting sensitivity to dexamethasone.

A growing number of studies have focused on the abnormal expression and activity of GR in steroid-resistant asthma. In our previous study (Zhang et al., 2019) and in this study, TNF-α did not alter the ratio of GRβ/GRα in airway epithelial cells. However, changes in the phosphorylation of GR as a consequence of activation may lead to many alterations in its function. Furthermore, the distribution of GR in cells is crucial for impaired GR nuclear translocation in some patients with severe asthma (Chang et al., 2015; Kobayashi et al., 2017). Consistent with previous studies (Zhang et al., 2019), the immunofluorescence results showed that Dex could induce GRα expression and nuclear transport, while TNF-α attenuated Dex-mediated induction of GRα nuclear translocation. However, despite TNF-α stimulation, nuclear translocation of GRα was not significantly affected in cells that were pretreated with Dex and R406.

Defective nuclear translocation of GR leads to reduced interactions with GREs. Additionally, the activity of the glucocorticoid-induced GRE reporter gene can be significantly inhibited by activated NF-κB (Chachi et al., 2017). Syk inhibition was reported to impair the phosphorylation of p65 NF-κB and the expression of NF-κB-dependent genes, such as IL-8 (Ashikawa et al., 2002; Takada and Aggarwal, 2004). This study showed that R406 alone did not attenuate allergen- or TNF-α-induced phosphorylation of p65 NF-κB but significantly enhanced Dex-mediated inhibition of p65 NF-κB activation, suggesting that in different cell types, Syk is involved in NF-κB signal regulation in different ways, but both factors showed inhibitory effects. Moreover, histone deacetylase (HDAC)2 activity is negatively correlated with steroid sensitivity, and its expression is reduced in severe asthma and chronic obstructive pulmonary disease (Hansbro et al., 2017; Palumbo et al., 2020). Increased NF-κB activity may regulate HDAC2 transcription (Ibi et al., 2017). In this study, no changes in HDAC2 expression were detected *in vitro*, nuclear level of HDAC2 may need to be detected However, in mouse models, Dex could restore HDAC2 activity when combined with R406. Thus, Syk may play a major role in the deficiencies in transcriptional corepressor expression and activity in severe asthma. On the other hand, GILZ, which is upregulated by GCs, plays an anti-inflammatory role by inhibiting the transcriptional activity that is dependent on AP-1 and NF-κB, and the expression of GILZ is downregulated by GR-Ser226 (Chachi et al., 2017; Marchini et al., 2019). We found that the expression of GILZ in airway epithelial cells induced by Dex alone was nearly 20 times that in the control group but continued to increase to 38 times after R406 pretreatment. That is, R406 could further promote GC-induced expression of GILZ, which was not affected by TNF-α. Another glucocorticoid-induced gene, GLCCI1, is considered to be a new pharmacogenetic determinant of asthma patient responses to ICS (Tantisira et al., 2011). Although the difference was not statistically significant at the time point analyzed, TNF-α inhibited and R406 promoted the expression of GLCCI1. It is thought that GLCCI1 regulates transactivation through its variants, rather than through gene and protein expression (Tantisira et al., 2011; Izuhara et al., 2014).

It is worth noting that Syk has good clinical prospects in the treatment of immunoinflammatory diseases. Multiple clinical trials have demonstrated that the small-molecule Syk inhibitors R406 and fostamatinib (R788, an oral prodrug metabolised to R406 *in vivo*) have desirable effects on rheumatoid arthritis and allergic rhinitis with few and acceptable side effects (Weinblatt et al., 2010; Kang et al., 2019; Wu et al., 2019). In December 2007, the inhaled agent R343 entered the first phase of clinical trials for the treatment of allergic asthma (Dimov et al., 2009), Regrettably, although shown to be relatively safe and well tolerated, R343 did not meet the primary or secondary endpoints in Phase 2 clinical study, with no difference between its ability to improve pre-bronchodilator FEV1 in 276 mild to moderate allergic asthma patients at 8 weeks and that of a placebo (ClinicalTrials.gov Identifier: NCT01591044). It is worth thinking whether other compounds or the indication changing from mild to moderate asthma to severe steroid-resistant asthma, will make it possible to treat asthma again with Syk inhibitors. Baluom and colleagues summarized the pharmacokinetics of R788 and R406 from three clinical studies (Baluom et al., 2013). Orally administered R788 is rapidly and completely hydrolyzed to R406, achieving peak plasma concentrations within 1–2 h. Across 80–600 mg doses, the half-life of R406 is between 13 and 21 h, which could potentially allow once or twice-daily dosing. In the body the majority of R406 attaches to plasma proteins reversibly, eventually reaches the organs and tissues. R406 underwent both glucuronidation and a CYP3A4-mediated para-O-demethylation, and 80% of the total drug was recovered in feces (Sweeny et al., 2010; Tang et al., 2022).

In addition, Syk inhibitor fostamatinib was approved for the treatment of chronic immune thrombocytopenia by the US Food And Drug Administration (FDA) in 2018 (Roskoski, 2020). Recently a multicenter Phase 2 clinical trial showed that fostamatinib may be promising therapeutic options for warm antibody autoimmune hemolytic anemia (Kuterf, et al., 2022), and is being further developed to treat renal transplantation (NCT03991780). Besides, fostamatinib is being evaluated for efficacy and safety in COVID-19 subjects in multiple clinical studies (NCT04579393, NCT04629703). Results of Phase 2 study proved that the addition of fostamatinib was safe and effective for improving clinical outcomes (Strich et al., 2021). The results of these clinical trials, which directly applied Fostamatinib to airway disorders, will be more valuable for clinical studies of fostamatinib or R406 in severe asthma in the future. All these clinical studies have shown that fostamatinib or R406 have generally manageable tolerability with no serious safety risks, so there is great potential for drug repurposing associated with Syk in severe asthma patients, but the target selectivity and effectiveness require further research. Currently, there is growing evidence demonstrating that some Chinese herbal ingredients are effective in treating asthma by suppressing the Syk-mediated inflammatory pathway (Ding et al., 2021; Yang et al., 2021). However, due to the unknown specificity of Syk inhibition, a large number of *in vivo* experiments are still needed.

Overall, we have demonstrated a new role for Syk inhibitors in the recovery of the anti-inflammatory effects of glucocorticoids on asthma. By combining R406 and dexamethasone, airway inflammation was significantly improved both *in vitro* and *in vivo*. Considering that Syk inhibitors have been used clinically to treat immune diseases, our study provides insight into the repurposing of Syk inhibitors for severe steroid-resistant asthma in the future.

## Data Availability

The raw data supporting the conclusions of this article will be made available by the authors, without undue reservation.
